# Magnesium Influences Membrane Fusion during Myogenesis by Modulating Oxidative Stress in C2C12 Myoblasts

**DOI:** 10.3390/nu13041049

**Published:** 2021-03-24

**Authors:** Monica Zocchi, Daniel Béchet, André Mazur, Jeanette A. Maier, Sara Castiglioni

**Affiliations:** 1 Department of Biomedical and Clinical Sciences L. Sacco, Università di Milano, Via G.B. Grassi 74, 20157 Milano, Italy; monica.zocchi@unimi.it (M.Z.); jeanette.maier@unimi.it (J.A.M.); 2 INRAE, UNH, Unitéde Nutrition Humaine, Université Clermont Auvergne, 63001 Clermont-Ferrand, France; daniel.bechet@inrae.fr (D.B.); andre.mazur@inrae.fr (A.M.); 3 Interdisciplinary Centre for Nanostructured Materials and Interfaces (CIMaINa), Università di Milano, 20133 Milan, Italy

**Keywords:** magnesium, myogenesis, oxidative stress, membrane fusion

## Abstract

Magnesium (Mg) is essential to skeletal muscle where it plays a key role in myofiber relaxation. Although the importance of Mg in the mature skeletal muscle is well established, little is known about the role of Mg in myogenesis. We studied the effects of low and high extracellular Mg in C2C12 myogenic differentiation. Non-physiological Mg concentrations induce oxidative stress in myoblasts. The increase of reactive oxygen species, which occurs during the early phase of the differentiation process, inhibits myoblast membrane fusion, thus impairing myogenesis. Therefore, correct Mg homeostasis, also maintained through a correct dietary intake, is essential to assure the regenerative capacity of skeletal muscle fibers.

## 1. Introduction

Magnesium (Mg) is the second most abundant cation within the intracellular compartment of the human body and is essential to all living cells, including skeletal myocytes [1,2,3]. Almost 25% of total Mg is contained in skeletal muscles where it plays a key role in myofiber relaxation by acting as a calcium (Ca) antagonist on Ca-permeable channels and Ca-binding proteins. It is known that a correct dietary Mg intake is positively associated with muscle strength and health [2] and that a Mg-deficient condition is associated with muscle cramps, spasms, weakness, and a higher risk of developing age-related sarcopenia [1,4]. Although the importance of Mg in the mature skeletal muscle is well established, little is known about the role of Mg in myoblasts and during myogenesis, i.e., process leading to muscle generation [5].

Skeletal muscle is a highly complex and heterogeneous tissue, the largest in the body. In the embryo, the first muscle fibers arise from the somites, transient structures which originate from the paraxial mesoderm. Additional fibers are generated from myogenic progenitors, which initially proliferate extensively and, once the muscle has matured, enter quiescence and reside as satellite cells between basal lamina and sarcolemma. Satellite cells use asymmetric divisions for self-maintenance and, at the same time, for generating a myogenic progeny with the potential to differentiate into new fibers. The differentiation process is coordinated by the sequential and coordinated expression of myogenic regulatory factors (MRFs), including MyoD and Myf5, which commit the cells to the myogenic program, and myogenin (Myog) and MRF4, which are responsible for the terminal stages of differentiation. Finally, differentiated myoblasts fuse with each other to form multiple nuclear myotubes [6,7]. In the adult, the satellite cells guarantee tissue renewal and repair.

Reactive oxygen species (ROS) are important players in the regulation of myogenesis [8,9]. ROS act in a hormetic fashion because they may exert beneficial or detrimental action depending on their amount and the duration of their production. A moderate increase of ROS is necessary for myogenesis. On the other hand, higher ROS levels can affect the efficiency of myogenic differentiation.

In this study we analyzed the effects of physiological (1 mM), low (0.1 mM) and high (3, 6, and 10 mM) extracellular Mg concentrations on myogenesis in C2C12 cells, an in vitro model of murine myoblasts which are able to efficiently differentiate in myotubes under specific culture conditions [10,11].

## 2. Materials and Methods

### 2.1. Cell Culture

C2C12 cells are murine proliferating myoblasts that, after serum depletion, differentiate to generate multinucleated myotubes [12]. The C2C12 cell line was purchased from American Type Culture Collection (ATCC). The cells were serially passaged in culture medium (CM) composed of Dulbecco’s Modified Eagle Medium (DMEM) containing high glucose and 20% of heat-inactivated fetal bovine serum (FBS), glutamine (2 mM), and 1% penicillin/streptomycin according to the manufacture’s instruction. To induce myogenic differentiation, 25,000 cells/cm^2^ were seeded. 24 h later, they were exposed to a differentiation medium (DM) consisting of DMEM with high glucose supplemented with 2% horse serum. In our experiments, the cells were cultured in DM containing low (0.1 mM), physiological (1 mM) or high (3, 6, and 10 mM) MgSO_4_ concentrations.

N-Acetylcysteine (NAC) (Sigma-Aldrich, St. Louis, MO, USA) was used to inhibit ROS production by pre-treating the cells for 24 h at the final concentration of 10 mM. The concentration was defined by performing a dose-response cell viability assay (data not shown). Images of cultured cells were acquired by optical microscopy with a Zeiss Primovert phase-contrast microscope (10× objective).

### 2.2. SDS-PAGE and Western Blot

C2C12 cells were lyzed in lysis buffer (50 mM Tris-HCl pH 7.4, 150 mM NaCl, 1% NP-40, 0.25% Na-deoxycholate) containing protease inhibitors. A syringe was used to better homogenize the lysates. Total proteins were quantified using the Bradford reagent (Sigma-Aldrich, St. Louis, MO, USA). Equal amounts of proteins were separated by SDS-PAGE on 4–20% Mini-PROTEAN TGX Stain-free Gels (Bio-Rad, Hercules, CA, USA) and transferred to nitrocellulose membranes by using Trans-Blot^®^ TurboTM Transfer Pack (Bio-Rad, Hercules, CA, USA). After blocking with bovine serum albumin (BSA), Western blot analysis was performed using primary antibodies against Myosin Heavy Chain (MHC) (R&D Systems, Minneapolis, MN, USA), Caveolin-3 (BD Biosciences, San Diego, California USA), Myomixer (R&D Systems, Minneapolis, MN, USA), and β-actin (Santa-Cruz Biotechnology, Dallas, TX, USA). After extensive washing, secondary antibodies conjugated with horseradish peroxidase (Amersham Pharmacia Biotech Italia, Cologno Monzese, Italy) were used. The immunoreactive proteins were detected with ClarityTM Western ECL substrate (Bio-Rad, Hercules, CA, USA) and images were captured with a ChemiDoc MP Imaging System (Bio-Rad, Hercules, CA, USA). Densitometry of the bands was performed with the software ImageLab (Bio-Rad, Hercules, CA, USA). The Western blots shown are representative and the densitometric analysis was performed on three independent experiments.

### 2.3. Immunofluorescence

The immunofluorescence staining and imaging were performed directly in culture wells. Cells were seeded in 24-well plates and, after 144 h of differentiation, fixed for 15 min in phosphate-buffered saline (PBS) containing 4% paraformaldehyde and 2% sucrose (pH 7.6). Cells were permeabilized and blocked for 30 min in a PBS solution containing 2% BSA and 0.3% Triton. To stain myotubes, the cells were incubated with anti-MHC primary antibody (R&D Systems, Minneapolis, MN, USA) and with an Alexa Fluor 488 secondary antibody (Thermo Fisher Scientific, Waltham, MA, USA). The nuclei were stained with 4′,6-diamidino-2-phenylindole (DAPI). Images were acquired using FLoid™ Cell Imaging Station (Thermo Fisher Scientific, Waltham, MA, USA).

### 2.4. ROS Production Analysis

For the detection of ROS [13], C2C12 cells were cultured in 96-well black plates (Greiner bio-one, Frickenhausen, Germany). At the end of the experiments, cells were incubated with 10 mM 2′-7′-dichlorofluorescein diacetate (DCFDA) (Thermo Fisher Scientific, Waltham, MA, USA) solution for 30 min at 37 °C. DCFDA was deacetylated by cellular esterases to a non-fluorescent compound which was then oxidized by ROS into the fluorescent molecule 2′,7′–dichlorofluorescein (DCF) (λexc = 495 nm, λemm = 529 nm). The dye fluorescent emission was measured using Varioskan LUX Multimode Microplate Reader (Thermo Fisher Scientific, Waltham, MA, USA). DCF fluorescence was normalized on cell counts. Images of DCF fluorescence emission were acquired on living cells cultured in 24-well plates. At the end of the experiment, cells were incubated for 30 min with the dye and images were captured with FLoid™ Cell Imaging Station (Thermo Fisher Scientific, Waltham, MA, USA). The results shown are the mean of three independent experiments performed in triplicate.

### 2.5. Statistical Analysis

Data are expressed as the mean ± standard deviation. The data were non-parametric and normally distributed and were analyzed using one-way ANOVA. The *p*-values deriving from multiple pairwise comparisons were corrected using the Bonferroni method. Statistical significance was defined as *p*-value ≤ 0.05. In the figures, * *p* ≤ 0.05; ** *p* ≤ 0.01; *** *p* ≤ 0.001; **** *p* ≤ 0.0001.

## 3. Results

### 3.1. Low and High Extracellular Mg Impair Myogenesis

**Figure 1:**
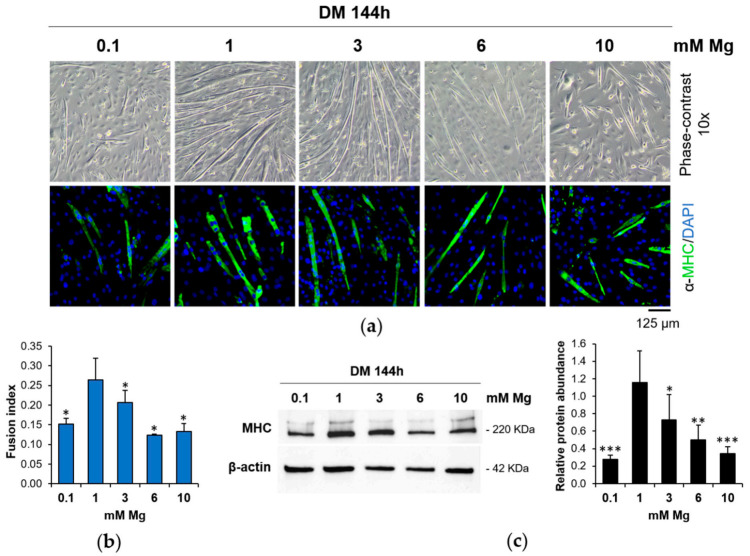
Low and high Mg concentrations inhibit myogenesis. C2C12 cells were cultured in differentiation medium (DM) for 144 h in the presence of different extracellular concentrations of Mg. (**a**) Pictures were taken with optical microscope (10× magnification, upper panels). After immunofluorescence with antibodies against Myosin Heavy Chain (MHC; green fluorescence), images were acquired using a fluorescence microscope (10× magnification, lower panels). The nuclei were stained with 4′,6-diamidino-2-phenylindole (DAPI). (**b**) Fusion index was calculated as the ratio of the number of nuclei within myotubes (>2 nuclei) to the total number of nuclei in the field and quantified based on (**a**). (**c**) MHC levels were analyzed by Western blot. β-actin was used as control of loading. A representative blot (left) and densitometry performed on three independent experiments and obtained by ImageLab (right) are shown. * Indicates significance with respect to 1 mM Mg (* *p* ≤ 0.05; ** *p* ≤ 0.01; *** *p* ≤ 0.001).

After 144 h of differentiation in low (0.1 mM), physiological (1 mM), and high (3, 6, and 10 mM) Mg concentrations, we analyzed C2C12 cells by optical microscope and by immunofluorescence using antibodies against Myosin Heavy Chain (MHC), a contractile protein only expressed in differentiated myotubes. As shown in Figure 1, we detected a significant reduction in the number of myotubes (Figure 1a) and in the fusion index values (nuclei in myotubes vs. total nuclei) (Figure 1b) both in low and high extracellular Mg vs. the control cells differentiated in physiological Mg concentrations. By Western blot (Figure 1c), we confirmed the reduction of the MHC expression in low and high Mg, suggesting that non-physiological extracellular concentrations of Mg impair myogenesis.

### 3.2. Low and High Mg Induce ROS Accumulation during Myogenesis

**Figure 2:**
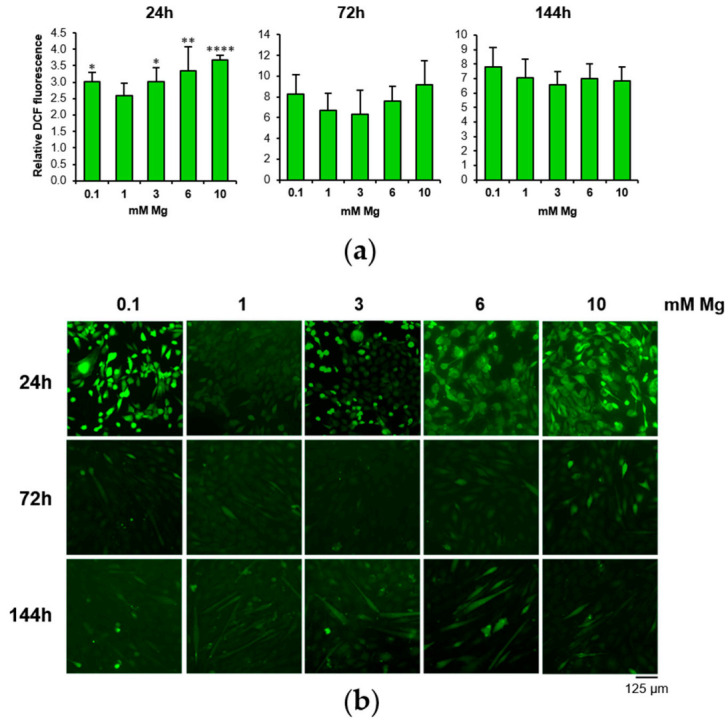
Low and high Mg induce ROS during myogenesis. After 24, 72 and 144 h of culture in DM in the presence of different extracellular concentrations of Mg, ROS accumulation was measured by 2′,7′–dichlorofluorescein (DCF) assay. Fluorescence was normalized to the cell number (**a**). Images of DCF fluorescence emission were acquired on living cells (10× magnification) (**b**). * Indicates significance with respect to 1 mM Mg (* *p* ≤ 0.05; ** *p* ≤ 0.01; **** *p* ≤ 0.0001).

Because ROS are critical signaling molecules involved in muscle differentiation [8], we measured their amounts by DCF assay during myogenesis of C2C12 cells in low- and high-Mg conditions. We found an increase of ROS after 24 h of serum deprivation both in low- and high-Mg conditions compared to the physiological condition, while we did not detect ROS modulation after 72 and 144 h of cell differentiation (Figure 2).

### 3.3. ROS Accumulation Is Involved in the Impairment of Myogenesis

**Figure 3:**
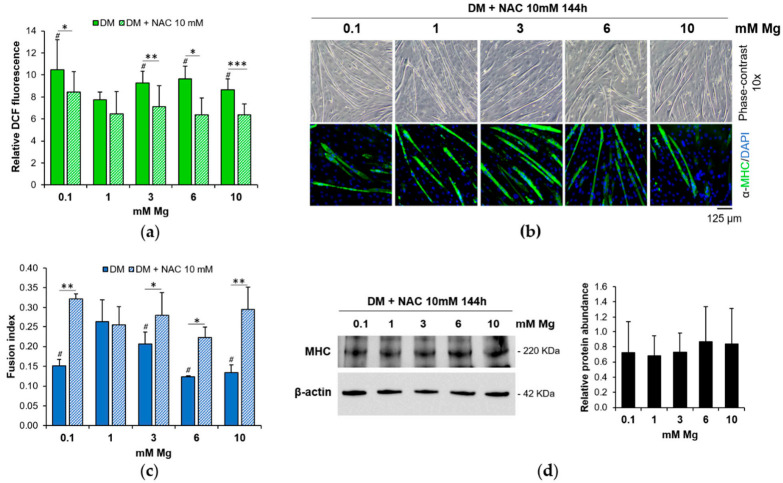
The antioxidant N-Acetylcysteine (NAC) prevents the impairment of myogenesis in low and high Mg. C2C12 cells were treated or not for 24 h with NAC in culture medium (CM) and then cultured in DM for 144 h with different extracellular concentrations of Mg. (**a**) ROS production was evaluated using DCF assay and the fluorescence was normalized to the cell number. (**b**) Upper panels: optical microscopy (10× magnification); lower panels: fluorescence microscopy after staining with antibodies against MHC (10× magnification). The nuclei were stained with DAPI. (**c**) Fusion index was quantified based on (**b**). (**d**) MHC total amounts were analyzed by Western blot. β-actin was used as control of loading. A representative blot (left) and the densitometry obtained by ImageLab (right) are shown. # Indicates significance with respect to DM 1 mM Mg (# *p* ≤ 0.05). * indicates significance between DM and respective DM + NAC 10 mM (* *p* ≤ 0.05; ** *p* ≤ 0.01; *** *p* ≤ 0.001).

To understand if the early ROS increase in low and high Mg is involved in the impairment of myogenesis, we analyzed the differentiation process after treating the cells with the antioxidant N-Acetylcysteine (NAC), a glutathione precursor, before inducing the cells to differentiate. By DCF assay, we demonstrated that NAC efficiently prevents ROS increase at 24 h of culture in low and high Mg (Figure 3a). After 144 h, the myotubes were analyzed by optical microscopy and by immunofluorescence of MHC. No significant differences were observed in C2C12 cells exposed to different Mg concentrations in the presence of NAC (Figure 3b). Moreover, after NAC pre-treatment, the fusion index values in low and high Mg were comparable to the controls (Figure 3c). Western blot in Figure 3d shows no appreciable differences in MHC total amounts in the presence of NAC under all the Mg conditions tested.

### 3.4. Low and High Mg Impair Myoblasts Fusion

**Figure 4:**
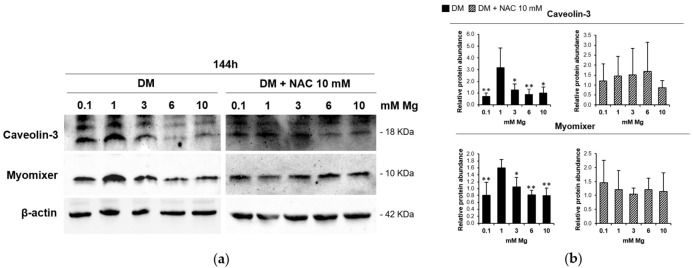
Low and high Mg inhibit myoblast fusion. C2C12 cells were treated or not for 24 h with NAC in CM and then cultured in DM for 144 h with different extracellular concentrations of Mg. Caveolin-3 and Myomixer expression was analysed by Western blot. β-actin was used as control of loading. A representative blot (**a**) and densitometry obtained by ImageLab (**b**) are shown. * Indicates significance with respect to 1 mM Mg (* *p* ≤ 0.05; ** *p* ≤ 0.01).

Because our data demonstrate that low and high Mg concentrations decrease the fusion index value, we investigated the levels of two key proteins involved in myoblast membrane fusion, Caveolin-3 and Myomixer, after 144 h of differentiation in the presence of different Mg concentrations. As shown in Figure 4, low- and high-Mg conditions downregulate both Caveolin-3 and Myomixer. Importantly, this effect is prevented by treating the cells with NAC.

## 4. Discussion

Magnesium is an essential mineral for human health and its homeostasis is regulated by the balance between intestinal absorption and renal excretion [14]. An insufficient dietary Mg intake, a long-term alcohol abuse, the chronic use of some drugs, and pre-existing pathologies, such as diabetes, can lead to hypomagnesemia [1]. On the other hand, patients with acute or chronic kidney disease, hypothyroidism, and especially cortico-adrenal insufficiency can develop hypermagnesemia [15]. Both hypomagnesemia and hypermagnesemia impact on cell, tissue, and organ functions, leading to many disorders, among which neuromuscular disorders. However, very little is known about the molecular effects of variations in Mg homeostasis on skeletal muscle cells.

Here we studied how low and high extracellular Mg concentrations impact on the process of muscle fiber formation. C2C12 murine myoblasts were induced to differentiate into myotubes using media with different Mg conditions. We demonstrate that low or high extracellular concentrations of Mg impair myogenesis because we observed a reduction in the number of myotubes and in the fusion index values compared to the physiological condition. We also detected an increase of ROS in low and high Mg during the early phase of the differentiation process, while after 72 and 144 h of differentiation, no differences in the amounts of ROS were found. We speculate that some antioxidants’ mechanisms are activated as an adaptive response to guarantee cell viability. It is known that ROS are implicated in different metabolic processes, including skeletal muscle differentiation. A moderate generation of ROS, in combination with growth factors and chemokines, is necessary for muscle regeneration and repair [16,17]. On the contrary, higher amounts of ROS might target mitochondria and mitochondrial DNA, inducing the block of myogenesis [8,18]. Moreover, higher ROS levels induce a depletion of intracellular glutathione which in turn further increases ROS accumulation. ROS increase induces NF-kB activation which contributes to lowering the expression of MyoD, thereby inhibiting myogenesis [19]. Coherently, we show that the ROS increase in low and high Mg is directly responsible for the impairment of myogenesis because the antioxidant NAC prevents the detrimental effect of low and high Mg on myogenesis, both in terms of myotube formation and fusion index. Moreover, after treatment with NAC, no significant differences in MHC expression were detected in all the Mg conditions tested.

Mg deficiency has been already demonstrated to induce oxidative stress in different cell types [20,21]. Interestingly, in myoblasts we also observed oxidative stress in high-Mg conditions. We can speculate that both low and high Mg could reduce the activity of some antioxidant enzymes, fundamental to maintaining ROS below a physiological level, and/or increase pro-oxidant molecules. Indeed, at low levels, ROS act as signaling molecules without exerting toxic effects, while at high levels they have detrimental effects.

To better understand the effects of extracellular Mg variations on the differentiation process, we focused on myoblast membrane fusion and detected a reduction in the amounts of two proteins involved in myoblast fusion, namely, Caveolin-3 and Myomixer. Indeed, lack of Caveolin-3 expression is sufficient to severely affect the fusion process during myogenesis [22,23]. Myomixer is a newly discovered muscle-specific membrane peptide with a fundamental role in the fusion pore formation [24,25]. Moreover, the antioxidant NAC rescues the levels of both Caveolin-3 and Myomixer in the cells induced to differentiate in low and high Mg. For this reason, we can assert that, in our model, ROS increase occurring in low- and high-Mg conditions impairs myogenesis by inhibiting the fusion process.

In conclusion, non-physiological extracellular Mg concentrations induce oxidative stress which affects myogenesis of C2C12 cells by inhibiting myoblasts’ fusion. Our data suggest that adequate Mg dietary intake and the maintenance of Mg homeostasis are necessary to guarantee correct skeletal muscle plasticity and the regenerative capacity of fibers, thus contributing to skeletal muscle health.

## Data Availability

The data presented in this study are openly available in Dataverse at https://dataverse.unimi.it/dataverse/nutrients/ (accessed on 1 March 2021).
